# Supplementary material for: Epigenetic Biomarkers and the Wnt/β-Catenin Pathway in Opisthorchis viverrini-associated Cholangiocarcinoma: A Scoping Review on Therapeutic Opportunities
Source: PLoS Negl Trop Dis. 2024 Sep 5;18(9):e0012477. doi: 10.1371/journal.pntd.0012477 (PMC11407677; doi:10.1371/journal.pntd.0012477)
Supplement: S1 Table — (DOCX) [file pntd.0012477.s001.docx]

**S1 Table.** The PCC (Population/Concept/Context) framework employed to structure key concepts, focusing on the crucial involvement of DNA methylation and histone modifications in Ov-CCA pathogenesis

| **PCC Elements** | **Definition** |
| --- | --- |
| Population | Individuals diagnosed with *Ov*-CCA; Individuals with opisthorchiasis without cancer diagnosis; Controls without opisthorchiasis or CCA diagnosis |
| Concept | Role of DNA methylation and histone modifications in *Ov*-CCA pathogenesis; Identification of epigenetically regulated genes and pathways in carcinogenesis; Potential use of abnormal methylation profiles as *Ov*-CCA biomarkers; Insights into therapeutic targets through epigenetic analysis |
| Context | Human observational studies from *O. viverrini* endemic regions in Southeast Asia; Tissue samples at different stages of disease progression; Emphasis on epigenetic studies measuring DNA methylation and histone modifications; Analysis and comparison of included studies clarifying relationships between epigenetic marks and *Ov*-CCA |
