# Supplementary material for: Epigenetic Biomarkers and the Wnt/β-Catenin Pathway in Opisthorchis viverrini-associated Cholangiocarcinoma: A Scoping Review on Therapeutic Opportunities
Source: PLoS Negl Trop Dis. 2024 Sep 5;18(9):e0012477. doi: 10.1371/journal.pntd.0012477 (PMC11407677; doi:10.1371/journal.pntd.0012477)
Supplement: S2 Table — (DOCX) [file pntd.0012477.s002.docx]

**S2 Table** The core methodologies frequently applied in *Ov*-CCA methylation studies

| Technique | Purpose/Description |
| --- | --- |
| Methylation-Specific PCR (MSP) | Detection of promoter methylation in specific genes |
| Bisulfite Sequencing Methods | Quantitative assessment of CpG methylation levels |
| Methylation Microarrays | Genome-wide DNA methylation profiling |
| Methylation-Sensitive High-Resolution Melting (MS-HRM) | High-throughput methylation screening |
| Immunohistochemistry | Evaluation of protein expression of genes of interest |
| RT-qPCR | Measurement of gene expression levels and validation |
| Bioinformatic and Statistical Analyses | Identification of significantly differentially methylated genes |
| Clinical Data Correlation | Association of methylation changes with clinical parameters |
| Functional Assays | Validation of functional effects of methylation changes |
