# Supplementary material for: Epigenetic Biomarkers and the Wnt/β-Catenin Pathway in Opisthorchis viverrini-associated Cholangiocarcinoma: A Scoping Review on Therapeutic Opportunities
Source: PLoS Negl Trop Dis. 2024 Sep 5;18(9):e0012477. doi: 10.1371/journal.pntd.0012477 (PMC11407677; doi:10.1371/journal.pntd.0012477)
Supplement: Attachment — Submitted filename: Response to the reviewers_PNTD-D-24-00341_R1.docx [file pntd.0012477.s003.docx]

**Journal Name**: PLOS Neglected Tropical Diseases

**Manuscript ID:**  **PNTD-D-24-00341**

**Title:** Epigenetic Biomarkers and the Wnt/β-Catenin Pathway in *Opisthorchis viverrini*-associated Cholangiocarcinoma: A Scoping Review on Therapeutic Opportunities

**Authors:** Alok Kafle, Sutas Suttiprapa, Mubarak Muhammad, Jan Clyden B Tenorio, Roshan Kumar Mahato, Norhidayu Sahimin, Shih Keng Loong

Dear Editor,

Thank you very much for your efforts on our manuscript, **PNTD-D-24-00341**. We are thankful for the reviewer’s critical comments and have revised the manuscript accordingly. I want to submit the revised manuscript to the PLOS Neglected Tropical Diseases. The responses to reviewer comments are summarized in this letter point-by-point in blue ink, and the corresponding revisions are highlighted in the revised manuscript.

We appreciate your precious time in reviewing our manuscript, and we would be very grateful if you would consider this manuscript for publication after review.

Yours sincerely,

Sutas Suttiprapa

**Reviewer #1**

: The authors conducted a systematic analysis of the available papers on the methylation state of individual genes in human samples from patients suffered from *Opisthorchis viverrini*-associated cholangiocarcinoma. The study was conducted to identify individual target genes for the subsequent development of gene therapy. The materials and methods are presented in clear and straightforward style. Nevertheless, I have suggestions for the Results section that would improve the manuscript since it requires major revision.

**Comment #1**

1. The lack of line numbering is unhelpful for the reviewer

**Response #1**

We apologize for any inconvenience caused, despite submitting the file in a numbered format. Thank you for your patience and for taking the time to review our paper.

**Comment #2**

2. In my opinion, the manuscript does not have enough examples of differences in hypermethylation of regulatory regions of individual genes compared to that in other types of cancer. I recommend indicating whether this is a characteristic feature of *Ov*-associated CCA or not. At the same time, the authors still provide such information about individual genes (Trop2 and RIZ1), but not for all genes. If there is no such information, then I recommend mentioning that there is no information.

**Response #2**

Thank you for your feedback on further contextualizing the epigenetic alterations observed in *Ov*-CCA relative to other cancers. We agree that this type of comparative analysis could provide valuable insights. However, performing an exhaustive literature search and data extraction for all genes across multiple cancer types would represent a significant expansion of the current review's aim, scope, and content.

As a scoping review, our objective is to provide a broad overview of the current state of research on epigenetic modifications in *Ov*-CCA, rather than an in-depth analysis of any single genes. While examples of differential methylation for a few genes were discussed to illustrate unique findings, a comprehensive gene-by-gene comparison was beyond the review's intended level of detail. Genes such as Trop2 and RIZ1 have been strongly implicated in upregulating key proteins and pathways crucial for cancer progression, such as Bcl-2 and Erα, respectively. Bcl-2 plays a critical role in inhibiting apoptosis, thereby promoting cancer cell survival. ERα and ERβ, on the other hand, facilitate estrogen-driven proliferation in hormone-sensitive cancers, supported by extensive research findings.

Due to feasibility limitations within the scoping review format, our focus was on synthesizing existing research specifically on *Ov*-CCA cancer pathogenesis and predicted pathways, with a comparison emphasis more limited to pathogenesis differences at the epigenetic level between *Ov*-CCA and other types of CCA. However, the lack of comparative data has been a significant limitation. We acknowledge that targeted investigations into similarities and distinctions across different cancer types would enhance our understanding of *Ov*-CCA biology. Nevertheless, we maintain that our scoping review has effectively mapped the current literature landscape and identified key priorities for future research studies.

**Comment #3**

3. The study also lacks a table that would summarize the most important findings for the characterization of *Ov*-CCA, in particular a table with examples of specific hypermethylated regions of genes unique to *Ov*-CCA would be useful.

**Response #3**

Thank you for highlighting the value of including a summary table in the review. You make an excellent point that consolidating key findings could help readers more easily identify distinguishing epigenetic characteristics of *Ov*-CCA versus other cancer types.

While the current main table presents information from the included studies' literature, a dedicated table focusing on comparisons would enhance accessibility and clarity on this important point. While acknowledging that such exploration would enhance our understanding of *Ov*-CCA pathogenesis and its implications for biomarkers and therapeutics, the scoping review format limits our ability to conduct an in-depth comparative analysis across other cancer types. Nevertheless, we have prepared a table for potential inclusion as a **supplementary file**, which we suggest the editor and reviewers to consider. Its relevance for inclusion in the manuscript will be determined based on their assessment. Thank you again for taking the time to provide this constructive criticism. Please let me know if you have any other feedback on how we could best address your point within the parameters of the scoping review methodology.

| **Features** | ***Ov*-CCA** | **Other Cancers** |
| --- | --- | --- |
| Most dysregulated pathway | Wnt/β-catenin | Varied - e.g., EZH2 inhibition suppressed Wnt/β-catenin in HCC |
| Unique methylation patterns | Trop2 hypermethylated and silenced | Trop2 generally overexpressed in other cancer types |
| Mutation profiles | Exon 6 TP53 mutations contributing to CCA | Mutation profiles are diverse and may vary between cancer types |
| Relationship between pathways | IL-6/STAT3 and Wnt pathways interaction via EBF1 | Not well explored in other cancers |
| Effect of pathways on progression | Wnt/β-catenin dysregulation linked to worse outcomes in CCA subtypes | Impact varies between cancer types |
| Cancer biomarkers | Hypermethylated genes show higher potential | Biomarkers depend on cancer driver genes and molecular alterations |
| Invasive/metastatic behavior | Promoter hypermethylation increases invasiveness | Mechanisms depend on cancer origins and stages |
| Response to targeted therapies | Wnt/β-catenin pathway inhibition shows promise | Response linked to genetic/epigenetic landscapes; trials ongoing |
| Immune system interaction | The hypermethylation and hypomethylation genes regulating inflammation, immunity, tumor microenvironment, DNA repair impact Wnt pathway | Dynamic, influenced by inflammation in tumor microenvironment |

**Comment #4**

3. It is necessary to write all genes and all proteins in accordance with the nomenclature rules for writing human genes (please refer to HUGO Gene Nomenclature Committee (HGNC).

**Response #4**

We appreciate the reviewer's insightful comment. Under **Table 1** summarizing the findings from the included (11) studies, we have formatted gene names alphabetically according to the guidelines set forth by the HUGO Gene Nomenclature Committee (HGNC), ensuring standardized nomenclature for human genes and gene products.

ALDH1A3 (Aldehyde Dehydrogenase 1 Family Member A3); CCND2 (Cyclin D2); DcR1 (Decoy Receptor 1); EBF1 (Early B-Cell Factor 1); EMP1 (Epithelial Membrane Protein 1); FILIP1L (Filamin A Interacting Protein 1-Like); HIC1 (Hypermethylated in Cancer 1); HOXA9 (Homeobox A9); HOXD9 (Homeobox D9); HTATIP2 (HIV-1 Tat Interactive Protein 2), also known as TIP30; IRF4 (Interferon Regulatory Factor 4); OPCML (Opioid Binding Protein/Cell Adhesion Molecule Like); PPP4C (Protein Phosphatase 4 Catalytic Subunit); PTEN (Phosphatase and Tensin Homolog); RASSF1 (Ras Association Domain Family Member 1); RUNX3 (Runt-Related Transcription Factor 3); SFRP1 (Secreted Frizzled Related Protein 1); SUZ12 (Suppressor of Zeste 12 Protein Homolog); TET1 (Ten-Eleven Translocation Methylcytosine Dioxygenase 1); TP53I3 (Tumor Protein P53 Inducible Protein 3); TROP2 (Tumor-Associated Calcium Signal Transducer 2); UCHL1 (Ubiquitin C-Terminal Hydrolase L1).

**Comment #5**

5. This review is not sufficiently illustrated. In my opinion, the manuscript lacks clear gene networks or crosstalks between signaling pathways to illustrate the main findings.

**Response #5**

You've raised an excellent point. As a review aiming to provide a high-level overview, visual representations could greatly aid comprehension and synthesis of key conclusions. In our scoping review, **Figure 3** helped identify pathways significantly enriched with the queried gene set, emphasizing those showing repeated Wnt signaling and higher combined scores for enhanced statistical significance and gene overlap confidence. Furthermore, the inclusion of the STRING network (Figure 4) in the main text serves to emphasize the identified crosstalk between dysregulated genes and signaling cascades such as Wnt/β-catenin. As a scoping review intended to offer a broad overview rather than an exhaustive analysis, generating new primary data illustrating molecular-level crosstalk between signaling pathways in the form of customized figures/tables exceeds its defined scope and purpose.

While the inclusion of existing pathway/network analyses in figure format may help communicate insights more accessible, developing new illustrations would extend beyond mapping the current research landscape. Conducting new primary data analysis and modeling was not within the scope of our scoping review methodology. Our aim was to systematically present and synthesize existing evidence as reported in individual studies. Nonetheless, we appreciate highlighting this as an area for potential enhancement in future-focused reviews on various possible targets emerging from our analysis.

Minor

1. page 29, 3rd paragraph – unreadable characters,

We didn’t find such characters. Please help us by being more specific to the lines or sentences.

2. The reference list is written in a different style and font

Updated to “Vancouver” style.

3. p.27, mutp53 should be decrypted the first time it appears in the text

*Line 604* A Mutant p53 (mutp53) protein can interact with components of the Wnt pathway, such as β-catenin, to enhance their activity.

**Reviewer #2**

This review identified epigenetic changes and Wnt/β-catenin pathway deregulation as key drivers in *Ov*-CCA pathogenesis. This study underscores the importance of the epigenetic modifications in *Ov*-CCA development, suggesting novel therapeutic targets within disrupted signaling networks. It is very interesting and valuable for therapy of Ov-CCA.

We appreciate the reviewer for recognizing the importance of our review and acknowledging the urgency of intervention strategies against Neglected Tropical Diseases (NTDs). We have revised the manuscript and incorporated the reviewer's suggestions to improve its quality.

**Reviewer #3:**

Reviewer's Responses to Questions

**Comment #1**

1. In Section “3. Results”, page 9.

“The initial search yielded 495 records from the four electronic databases.”

In Figure 1. Flowchart depicting the identification and selection process of studies, page 10.

“Records identified through database search (n=1050)”

The number “495 records” and “n = 1050”, which number is an initial searching record?

Thank you for the queries.

**Response #1**

We apologize for the oversight. As stated in the manuscript, we included all studies investigating epigenetic mechanisms in the context of *O. viverrini*-related CCA and non-*O.viverrini* induced CCA. Initially, our search returned 1050 results (as shown in the compiled figures below). Subsequently, we refined our search by employing broad keywords to focus on the pertinent studies. We are grateful to the reviewer for highlighting this typographical error. The appropriate changes have been made to n = 1050.

**Comment #2**

2. In Section “3.1 Sample Size and Techniques”, page 10 “, immunohistochemistry (IHC), quantitative PCR (qPCR), and comprehensive computational analysis (supplementary 2).”

Is Supplementary 2 referred to in Supplementary Table 2?

**Response #2**

Thank you for bringing this to our attention. We acknowledge the oversight regarding the reference to "supplementary 2" in Section 3.1. Upon review, it should correctly refer to "**Supplementary Table 2**." We apologize for any confusion this may have caused and appreciate your understanding.

**Comment #3**

3. In “TP53 Mutations and EBB2 Amplification in Ov-CCA”, page 15

Is EBB2 the same gene as (ERBB2) described in the text?

**Response #3**

Yes, EBB2 is referring to the same gene as ERBB2 described in the text. ERBB2 is the official gene symbol and name for the HER2/neu receptor protein-coding gene. ERBB2 stands for "Erb-B2 receptor tyrosine kinase 2". It is also commonly called HER2 (Human Epidermal Growth Factor Receptor 2). The passage mentions ERBB2 gene amplification in clusters of liver disease related to fluke infection. It then refers to the HER2 receptor being encoded by the ERBB2 gene. Hence, EBB2 is simply another way of writing out the full gene name of ERBB2/HER2. It is referring to the same gene that encodes the HER2 receptor protein.

However, "EBB2" is a non-standard abbreviation and appears less preferred compared to "ERBB2." While we found a few instances of its use in the literature (references below), for consistency throughout this paragraph, we have opted to use "ERBB2" as the appropriate term. Hence, we agree with the reviewer's concern and prefer using ERBB2 throughout rather than switching between ERBB2 and EBB2.

https://doi.org/10.1016/j.clinimag.2020.11.024

doi: [10.18632/aging.102079](https://doi.org/10.18632%2Faging.102079)

<https://cancertools.org/cell-lines/mcf10a-her2-157986/>

**Comment #4**

**4. In Section “5. Conclusion and perspectives:”, page 31**

The authors mentioned, “Unlike genetic mutations, epigenetic alterations are reversible and dynamically influenced by the tumour microenvironment, making them attractive candidates for minimally invasive testing and disease progression tracking.”

How does the tumor microenvironment influence epigenetic alterations? Or what evidence support this tumor microenvironment-related epigenetic changes?

**Response #4**

Thank you for your feedback and inquiry. You have made a valid point that the lack of strong evidence on tumor microenvironment on epigenetic changes has not yet reached the evidential threshold for definitive confirmation. However, some studies support the notion that the tumor microenvironment plays a significant role in shaping epigenetic alterations in cancer cells. Some of the findings are mentioned below:

- Hypoxia/low oxygen levels within the tumor microenvironment have been shown to induce widespread changes in DNA methylation and histone modifications in cancer cells. The tumor microenvironment, particularly hypoxia mediated by HIF-1α activation, influences epigenetic alterations such as intragenic DNA methylation. These alterations contribute to changes in alternative splicing patterns observed in cancer cells, potentially impacting tumor aggressiveness and patient outcomes (1).
- Inflammatory cells and cytokines in the microenvironment can induce epigenetic changes. In hepatocellular carcinoma (HCC), tumor-associated macrophages (TAMs) undergo epigenetic modifications influenced by the tumor microenvironment, including hypoxia and cytokine signals. These changes alter TAM polarization (M1/M2 phenotype), affecting their immunomodulatory functions and promoting tumor progression, drug resistance, and immune evasion. Epigenetic regulation of TAMs also impacts their secretion of cytokines and enzymes like MMP-9, which is crucial for angiogenesis and extracellular matrix remodeling in HCC metastasis and invasion (2).
- Mutations in metabolic enzymes, such as isocitrate dehydrogenases (IDHs), can produce oncometabolites like D-2-hydroxyglutarate (D-2HG), which disrupt cellular metabolic processes and contribute to tumorigenesis. Furthermore, the altered metabolic profile of tumors creates a hostile microenvironment characterized by low glucose, high lactate levels, and hypoxia. These conditions sustain tumour growth and influence immune cell functionality within the tumor microenvironment (TME). In the TME, the competition for nutrients, particularly glucose, between tumor cells and immune cells, such as T cells, plays a crucial role in modulating immune responses. Tumor cells avidly consume glucose, limiting its availability for T cells and impairing their effector functions. This metabolic competition contributes to T cell dysfunction, characterized by reduced cytokine production (e.g., interferon-gamma) and impaired anti-tumor immune responses (3). Glycolytic enzymes like glyceraldehyde-3-phosphate dehydrogenase (GAPDH) and metabolic sensors like AMP-activated protein kinase (AMPK) are involved in these metabolic adaptations and can influence T cell activation and function under nutrient-deprived conditions.
- Cancer-associated fibroblasts communicate with cancer cells through extracellular vesicles, transporting non-coding RNAs that regulate recipient cell epigenetics. Extracellular vesicles (EVs), mediated by cancer-associated fibroblasts (CAFs), facilitate epigenetic modifications that promote therapy resistance and tumor progression, demonstrating their critical involvement in shaping the adaptive tumor microenvironment (4).

Therefore, in summary, multiple components of the complex tumor microenvironment, including hypoxia, inflammation, extracellular signaling and metabolic stresses, can elicit epigenetic changes that may contribute to phenotypic heterogeneity and impact cancer progression. This supports the claim that epigenetic mechanisms are dynamically involved in influencing the tumor microenvironment.

1. doi: [10.1007/s12038-019-9977-0](https://doi.org/10.1007%2Fs12038-019-9977-0)
2. doi: [10.1002/cam4.6717](https://doi.org/10.1002%2Fcam4.6717) , <https://doi.org/10.1038/s41423-021-00756-y>
3. [doi: 10.1016/j.chembiol.2023.08.011](https://doi.org/10.1016/j.chembiol.2023.08.011)
4. doi: [10.3390/cancers12082324](https://doi.org/10.3390%2Fcancers12082324)

--------------------

<b>Summary and General Comments</b></br></br>

Use this section to provide overall comments, discuss strengths/weaknesses of the study, novelty, significance, general execution and scholarship. You may also include additional comments for the author, including concerns about dual publication, research ethics, or publication ethics. If requesting major revision, please articulate the new experiments that are needed.
